# Interaction between the *TCF7L2* gene and dietary intake on metabolic syndrome risk factors among Saudi Arabian adults

**DOI:** 10.3389/fnut.2025.1513088

**Published:** 2025-03-18

**Authors:** Maha S. Al-odinan, Najlaa M. Aljefree, Noha M. Almoraie, Marwan A. Bakarman, Hani A. Alhadrami, Israa M. Shatwan

**Affiliations:** ^1^Food and Nutrition Department, Faculty of Human Sciences and Design, King Abdulaziz University, Jeddah, Saudi Arabia; ^2^Family and Community Medicine Department, Rabigh Faculty of Medicine, King Abdulaziz University, Jeddah, Saudi Arabia; ^3^Department of Medical Laboratory Sciences, Faculty of Applied Medical Sciences, King Abdulaziz University, Jeddah, Saudi Arabia; ^4^Molecular Diagnostic Lab, King Abdulaziz University Hospital, King Abdulaziz University, Jeddah, Saudi Arabia; ^5^King Fahad Medical Research Center, King Abdulaziz University, Jeddah, Saudi Arabia

**Keywords:** *TCF7L2*, total energy intake, saturated fatty acid, waist circumference, insulin, Saudi, adult

## Abstract

**Introduction:**

Transcription factor-7-like 2 (*TCF7L2*) is the most critical type 2 diabetes (T2D) gene identified to date. The single-nucleotide polymorphism (SNP) rs7903146 in *TCF7L2* in T2D interacts with dietary factors; however, research on nutrigenetics among Saudi Arabians is limited. This study investigated the interaction between the SNP rs7903146 and dietary intake on factors that may contribute to MetS among Saudi Arabian adults.

**Methods:**

This cross-sectional study included 271 adult participants (aged 20–55 years) of both genders with or without overweight or obesity (body mass index between 18–35 kg/m^2^). Anthropometric measurements and dietary assessments using a food frequency questionnaire were performed. Fasting blood samples were collected to analyze serum lipid, glucose, and insulin levels. Genetic analysis was performed using real-time polymerase chain reaction. Univariate regression was used to examine the association between the *TCF7L2* SNP rs7903146 and laboratory parameters, and to test SNP-diet interactions. The additive model was used in the analysis and the T allele was the effect allele.

**Results:**

A marginal significant association was observed between SNP rs7903146 and waist circumference (WC) (*p* = 0.05). Carriers of TT genotype had the highest WC (83.5 ± 20.1 cm), when compared with the CC genotype (80 ± 14.2 cm) and the TC genotype (77.9 ± 13.9 cm). The SNP rs7903146 was significantly associated with total energy intake (*p* = 0.04) and saturated fatty acids (SFA, *p* = 0.005), and TT carriers had the highest total energy and SFA consumption (3606.9 ± 1554.7 kcal, 66.8 ± 52.0 g, respectively). Only one near significant interaction was observed between SNP rs7903146 and total energy intake on insulin levels (*p* = 0.04), with carriers of the TT genotype showed a greater reduction in insulin values (−5.3 ± 3.5) at lower energy intake when compared with the CC (−2.4 ± 3.1), and TC (−4.7 ± 2.8). No significant interaction was found.

**Conclusion:**

The present study observed significant associations between SNP rs7903146 and total energy and SFA consumptions. The TT carriers had increased consumption of total energy and SFA. Future studies using larger sample sizes are required to confirm significant interaction between SNP rs7903146 and diet on factors that may contribute to MetS in the Saudi population.

## 1 Introduction

Metabolic syndrome (MetS) comprises a cluster of metabolic abnormalities occurring together, including insulin resistance, dyslipidemia, abdominal obesity, and hypertension, which increase the risk of type 2 diabetes mellitus (T2D), cardiovascular disease (CVD), and atherosclerosis (1). The prevalence of MetS among the Saudi Arabian population is 31.6% according to the International Diabetes Federation criteria and 39.9% according to the ATP III criteria (2). The significant increase in the prevalence of MetS is an important illustration of the complex interplay of genetic and environmental factors on disease progression (3, 4). Genome-wide association studies have identified genetic variants in the Wnt signaling pathway related to T2D, of which transcription factor-7-like 2 (*TCF7L2*) has been reported as one of the genes most significantly associated with T2D risk (5, 6). Additionally, *TCF7L2* is associated with MetS (7), dyslipidemia (7–9), and obesity markers (10, 11).

As part of the Wnt signaling pathway, TCF7L2 is activated by Wnt ligands or certain growth factors, including insulin. It plays a vital role in regulating different biological processes, including gluconeogenesis, and improves lipid accumulation in various organs, such as the liver and adipose tissues (12). Notably, the single-nucleotide polymorphism (SNP) rs7903146 (C/T) in *TCF7L2* has been demonstrated to be one of the strongest genetic risk factors for T2D in different ethnic groups (13–17) as well as different features of MetS (7, 8, 10). Only two studies were performed in Saudi Arabia to study the relationship between rs7903146 (C/T) and T2D. The first was an earlier case–control study of 1,166 patients with T2D and 1,235 healthy volunteers, which found a significant association between *TCF7L2* SNP rs7903146 and T2D, where carriers of the T allele had a 1.55-fold increased risk of T2D compared to C allele carriers (18). The second was a case–control study that included 359 patients with T2D and 351 healthy controls. It did not report any association between T2D risk and the *TCF7L2* SNP rs7903146 but with other SNPs, rs12255372 and rs4506565 (19).

Findings from the Diabetes Prevention Program and Diabetes Prevention Study reported that interactions with dietary factors can modulate the association between *TCF7L2* variants and diabetes risk (20, 21). Several studies have been conducted in the field of nutrigenetics, albeit with inconsistent findings (22–27). Furthermore, a high intake of saturated fatty acids (SFA) increases the risk of MetS (22). An interventional study showed that low-fat and high-carbohydrate diets have favorable effects on weight loss in individuals carrying the TT genotype in *TCF7L2* (23). Whole grains and dietary fiber interact with *TCF7L2* variants in T2D risk (24–26). However, meta-analyses could not replicate these significant interactions (27). Saudi Arabia has distinct eating habits that are shaped by traditional meals, consumption of foods high in energy, and increasing dependence on processed foods and consumption of sugar-sweetened beverages (28). Overall, the eating habits of the Saudi population have changed significantly. Meat, sugar, animal fat, and dairy product consumption has increased (29); conversely, fruit and vegetable consumption varies by location and demographic group. Such changes are linked to economic growth, urbanization, and shifting lifestyles in the wake of globalization (30). The eating patterns could significantly affect health outcomes, especially with regard to metabolic disorders. Saudi Arabia is among the top 20 countries with the highest rates of diabetes per capita, with an average frequency of 15% (31). Diet is one of the main modulatory factors associated with the effect of the *TCF7L2* SNP rs7903146, but studies analyzing gene-diet interaction that may contribute to the development of the metabolic diseases such as T2D are lacking in Saudi Arabia. Therefore, the present study aimed to investigate the relationship between the *TCF7L2* SNP rs7903146, dietary characteristics and factors that may contributing to MetS among adult population of Jeddah Saudi Arabia. The specific objectives of this study were to: (1) assess the association between the SNP rs7903146 and factors that may contribute to MetS, among Saudi adults, and (2) to investigate the interaction between the SNP rs7903146 and dietary characteristics, on factors that may contribute to MetS, among Saudi adults.

## 2 Methods

### 2.1 Study participants

A cross-sectional study was conducted with participants from Jeddah, Saudi Arabia, recruited between January 2023 and November 2023. The study sample included 274 adults (aged 20–55 years) of both genders with or without overweight or obesity according to body mass index (BMI ranged 18–35 kg/m2). Exclusion criteria included the presence of any diagnosed disease (such as both type 1 and 2 diabetes, CVD, hypertension, human immunodeficiency virus infection, liver disease, renal failure, cancer, or gastrointestinal disorder), use of medication that affected serum lipids or diabetic parameters, chemo or radiation therapy, surgery within the last 6 months, and women who were pregnant or lactating.

Study participants were recruited at the King Fahd Medical Research Center after having received the invitation extended to all students and personnel of King Abdulaziz University via the university’s email and social media platforms. The invitation included a brief description of the study, the purpose and the inclusion criteria for voluntary participation. Participants visited the Nutrition Assessment Lab, Faculty of Human Sciences and Development, and Roya Labs, Specialized Medical Laboratories, at King Abdulaziz University. Factors contributing to MetS assessed included BMI, waist circumference (WC), hip circumference (HC), systolic (SBP) and diastolic blood pressure (DBP), total cholesterol (TC), low-density lipoprotein cholesterol (LDL), high-density lipoprotein cholesterol (HDL) and triglycerides (TG), glucose, and insulin while dietary factors included total energy and macronutrient intake.

Ethical approval was obtained from the Biomedical Ethics Research Committee of King Abdulaziz University (reference no. 269-22). All subjects signed the informed consent for participation and consent for genetic analysis.

### 2.2 Study protocol

#### 2.2.1 Sociodemographic and lifestyle questionnaire

A questionnaire was prepared to collect sociodemographic, anthropometric, lifestyle and dietary data through face-to-face interviews with the participants. Sociodemographic and lifestyle data included age, gender, education level, employment, marital status, household income, smoking status, and self-reported physical activity level (i.e., sedentary; light activity, less than half an hour 2 days or less per week; moderate activity, at least one hour 3–4 days per week; or vigorous activity, more than one hour 5 days or more per week).

#### 2.2.2 Anthropometric measurements

Anthropometric assessments were performed by one of the research teams at the beginning of the visit, before collecting blood samples from the participants. Height was measured using an automatic stadiometer (Detector Weigh Beam Eye-Level Scale, United States). Weight was measured using an electronic weighing scale (Yunmai Mini, China) with participants barefoot, wearing light clothing and with an empty bladder. BMI was calculated as the body weight in kilograms divided by the height in meters squared (kg/m^2^). WC was measured at the midpoint between the lower border of the rib cage and the iliac crest using a non-elastic tape measure. Additionally, the HC was measured in the distance around the largest part of the hips and the widest part of the buttocks. Blood pressure was measured while the participants were sitting (N83, TMB-1583-S, Nahdi Company).

#### 2.2.3 Dietary intake assessment

Dietary habits were assessed using a validated food frequency questionnaire (FFQ) (32) to estimate participants’ energy and nutrient intake. The FFQ consisted of 135 items divided into eight groups. Participants were asked to recall and estimate their food consumption patterns over the past year using a range of frequency options (never, 1–3 per month, once per week, 2–4 per week, 5–6 per week, once per day, 2–3 per day, 5–6 per day, 6+ per day). The Photographic Atlas of Food Portions developed by the Ministry of Health in Abu Dhabi, UAE (33), was used to help participants estimate the quantity of food consumed. Daily food intake was calculated from the FFQ according to the following formula: frequency of intake (conversion factor) × weight of food consumed (34), according to data from the Atlas. The FFQs were analyzed using Nutritionist Pro software to obtain the calories and macronutrients. Moreover, the nutritional composition of specific Arabian dishes, such as meat and chicken kabssah, shawarma, and Arabic coffee, was obtained as previously reported (35), and the composition of camel meat food was obtained as reported elsewhere (36).

### 2.3 Biochemical analysis

The participants visited Roya Labs at King Fahd Medical Research Center after 12 h of fasting. Fasting blood samples were collected via venipuncture and placed into gel tubes (gold for lipid analysis) and sodium fluoride tubes (gray for glucose analysis). Plasma was separated via centrifugation (5,000 × *g* for 3 min at 4°C), and the resulting samples were transferred to Eppendorf tubes and stored at −40°C for subsequent analyses. Serum lipids, including TC, LDL, HDL, TG, and glucose, were analyzed at Tibyana Medical Lab, Jeddah, Saudi Arabia, using a Beckman Coulter analyzer (Model DXC700 AU). This instrument uses an enzymatic method to measure glucose and lipid levels. Insulin levels were analyzed using an enzyme-linked immunosorbent assay (Thermo Fisher Scientific) in the Biochemistry unit at the King Fahd Medical Research Center.

### 2.4 DNA isolation and *TCF7L2* SNP rs7903146 genotyping

Blood samples for genetic analysis were collected from EDTA-containing samples. Genetic analyses were performed by Haven Scientific Company. Briefly, genomic DNA was extracted from the buffy coat of white blood cells using spin-column-based technology (Haven Science Kit) at the Biochemistry Unit of King Fahd Medical Research Center. The spin column-based DNA extraction method comprises four stages: cell lysis, binding of the nucleic acid to the silica gel membrane, washing of the nucleic acid bound to the silica gel membrane, and elution of the nucleic acid (37). The DNA samples were then stored at −20°C. The samples were then transferred to Haven Science for genotyping. The SNP rs7903146 in *TCF7L2* (Haven Scientific, Saudi Arabia; Catalog Number PCR-SNP-RS7903146-11-150) was genotyped using real-time polymerase chain reaction (PCR). The qPCR SNPs were purchased from Haven Scientific. For gene amplification, a Probe Multiplex Real-Time PCR Master Mix (Haven Scientific, PCR6905) was used according to the manufacturer’s protocol. Briefly, 5 μL of the Probe Master Mix was mixed with 1 μL of the SNP assay, 1 μL of DNA, and 3 μL of RNase-free water and placed in 0.2-mL qPCR 96-well plate, semi-skirted (Haven Scientific, PCR-SSP-02). The plates were sealed with a Real-Time PCR Optical Adhesive Seal (Haven Scientific, PCR-OS-0011). The plates were run on a QuantStudio^™^ 5 Real-Time PCR System, 96-well, 0.2 mL (A28139, Applied Biosystems) using the following protocol: 3 min at 95°C, followed by 40 cycles of 95°C for 15 s and then 60°C for 60 s (data collection). Fluorescence from the FAM channel corresponded to the wild-type allele, whereas fluorescence from the VIC channel corresponded to the SNP allele. Fluorescence data were analyzed, plotted, and viewed using TaqMan Genotyper Software V1.7.1 (Applied Biosystems).

### 2.5 Statistical analysis

Statistical analyses were performed using SPSS software version 29. Descriptive statistics for continuous variables were presented as means and standard deviations. Differences in variables between genders were analyzed using the chi-square test. Normal distribution was checked for continuous variables, and log_10_ transformation was applied to skewed data based on the Shapiro–Wilk test. Continuous variables, including dietary intake, were analyzed using independent t-tests. The agreement with Hardy–Weinberg equilibrium expectations was tested using a χ^2^ goodness-of-fit test (*p* > 0.05). Additive models were used, given the sufficient frequency of rare homozygotes. Univariate regression was used to examine the association between the *TCF7L2* SNP rs7903146 and factors that may contribute to MetS, and dietary intake. The SNP-diet interactions on factors that may contribute to MetS were tested by including the interaction term in the univariate regression models. The calories from total energy, carbohydrate, fat, SFA, monounsaturated fatty acid (MUFA) and polyunsaturated fatty acids (PUFA) were used in the interaction analysis. The models were adjusted for age, sex, smoking status, physical activity, and BMI (continuous). *p*-values <0.05 indicated association. Multiple testing correction using the Bonferroni method was applied to test interaction effects (0.05/11 factors that may contribute to MetS x 6 nutrients, including total calories = adjusted p-value of 0.0007 ≈ 0.001).

Power analysis was performed based on Cohen’s *F*^2^ for gene-diet interactions in a regression model (38). The formula used was as follows:

The study was powered at 80% to identify SNPs with a Minor Allele Frequency = 0.39, and our sample size was 264, and the significance level was set at 0.0007.

N = sample size (264). $n=\frac{{\left(Z_{1-\alpha }\pm Z_{1-\beta }\right)}^{2}}{F^{2}}$

Z_1-α/2_ = the significant level (α = 0.007, Z_1-α/2_ = 3.42).

Z_1-*β*_ = the desire power 80% (Z_1-β_ = 0.84).

*F^2^ =* effect size.

Therefore, our study was able to detect an effect size = 0.0646 corresponding to an *R^2^* value of approximately 6.5% of the variance explained by the interaction term. The current study was powered to detect low (0.02, 2% variance explained) to moderate (0.15, 15% variance explained) interaction effects.

## 3 Results

The demographic, anthropometric and laboratory characteristics of the study participants, stratified by sex are presented in Table 1. Three participants did not have genetic data; therefore, 271 were included in the association analysis. Another three participants had an incomplete FFQ, so only 268 were included in the interaction analysis. There were statistically significant differences between sexes in most characteristics, including age, marital status, education level, employment, smoking status, and physical activity. In particular, women were older than men (28.1 ± 7.6 years vs. 24.1 ± 7.1 years, *p* < 0.001). Most participants were single (67.3% female and 88% male), undergraduates (64% females and 94.4% males), and students (57.6% females and 78.8% males). Additionally, the number of non-smokers and past smokers was higher in females than in males (*p* < 0.001). Furthermore, most participants practiced light physical activity (40.1% of females and 38.8% of males. However, male participants tended to engage in moderate and vigorous physical activity than female participants (33.7 and 16.3% vs. 19.8 and 7.2%, respectively) (*p* < 0.001). Moreover, statistically significant differences were found in anthropometric (BMI, WC, HC) and blood pressure (SBP, DBP) measurements between the sexes (*p* < 0.001). All these parameters were higher in males than in females. Most laboratory parameters (TC, HDL, TG, glucose, and insulin) indicated statistically significant differences between sexes. In particular, females showed higher levels of TC and HDL than males, while males showed higher levels of TG, glucose, and insulin than females.

**Table 1:** Characteristics of the study participants stratified by sex.

Characteristics	Male (*n* = 101)	Female (*n* = 173)	*p* value
Age (years)	24.1 ± 7.1	28.1 ± 7.6	<0.001
Marital status (*n*, %)			
Single	89 (88.1)	117 (67.6)	<0.001
Married	12 (11.9)	47 (27.2)	
Divorced	0	9 (5.2)	
Education level (*n*, %)			
High school or low	3 (3.0)	1 (0.6)	<0.001
Undergraduate	96 (95.0)	111 (64.2)	
Postgraduate	2 (2.0)	61 (35.3)	
Employment (*n*, %)			
Student	79 (78.2)	100 (57.8)	0.001
Employee	18 (17.8)	50 (28.9)	
Unemployed	4 (4.0)	23 (13.3)	
Smoking status (*n*, %)			
Non-smoker	57 (56.4)	151 (87.3)	<0.001
Current smoker	40 (39.6)	15 (8.7)	
Former smoker	4 (4.0)	7 (4.0)	
Household income (*n*, %)			
Less than 5,000 SAR	4 (4.0)	9 (5.2)	0.11
5,000–10,000 SAR	23 (22.8)	60 (34.7)	
10,000–20,000 SAR	40 (39.6)	47 (27.2)	
More than 20,000 SAR	34 (33.7)	57 (32.9)	
Physical activity (*n*, %)			
Sedentary	14 (13.9)	61 (35.3)	<0.001
Light activity	38 (37.6)	67 (38.7)	
Moderate activity	33 (32.7)	33 (19.1)	
Vigorous	16 (15.8)	12 (6.9)	
BMI (kg\m^2^)	27.5 ± 7.4	24.0 ± 6.0	<0.001
Waist circumference (cm)	88.8 ± 16.6	74.4 ± 11.8	<0.001
Hip circumference (cm)	108.2 ± 14.6	100.4 ± 14.4	<0.001
Systolic blood pressure (mmHg)	138.4 ± 13.5	119.9 ± 13.1	<0.001
Diastolic blood pressure (mmHg)	81.3 ± 9.8	71.4 ± 8.8	<0.001
Total cholesterol (mg\dl)	167.4 ± 31.6	176.9 ± 36.1	0.02
High-density lipoprotein cholesterol (mg\dl)	44.0 ± 9.0	53.9 ± 10.7	<0.001
Low-density lipoprotein cholesterol (mg\dl)	108.3 ± 26.1	110.4 ± 28.9	0.62
Triglycerides (mg\dl)	72.1 ± 31.1	62.6 ± 24.8	0.01
Glucose (mg\dl)	88.6 ± 20.6	83.8 ± 9.8	0.01
Insulin (μIU/mL)	18.8 ± 15.4	11.39 ± 8.5	<0.001

Data are presented as mean ± SD for continuous variable and number and frequency (%) for categorical variables. *P* value was tested using Chi square for categorical variables and independent *t*-test for continuous variables.

Table 2 shows the dietary intake of study participants by sex. Males showed significantly higher intake of total energy, protein, carbohydrate, total fat, cholesterol, SFA, MUFA, PUFA, omega-3, omega-6 (*p* < 0.001), and dietary fiber (*p* = 0.005) than females.

**Table 2:** Dietary intake of participants by sex.

Nutrients	Male (*n* = 101)	Female (*n* = 173)	*P* value
Energy (calorie)	4235.1 ± 1425.2	2947.7 ± 1172.1	<0.001
Energy (kilojoules)	17668.6 ± 5949.2	12313.6 ± 4901.2	<0.001
Protein (g)	431.2 ± 557.3	192.1 ± 232.1	<0.001
Carbohydrates (g)	468.2 ± 157.3	340.1 ± 134.1	<0.001
Total fat (g)	197.4 ± 117.2	119.6 ± 65.1	<0.001
Cholesterol (g)	918.6 ± 557.8	522.4 ± 307.9	<0.001
Saturated fatty acid (g)	73.1 ± 50.8	43.4 ± 26.2	<0.001
Monounsaturated fatty acid (g)	56.3 ± 22.9	36.6 ± 18.2	<0.001
Polyunsaturated fatty acid (g)	37.0 ± 17.2	22.3 ± 11.6	<0.001
Omega-3 (g)	1.2 ± 0.8	0.7 ± 0.7	<0.001
Omega-6 (g)	7.1 ± 4.7	4.1 ± 3.7	<0.001
Total dietary fiber (g)	31.3 ± 13.9	26.5 ± 13.1	0.003

Data are presented as mean ± SD. *P* value was tested using independent *t*-test.

**Figure 1:**
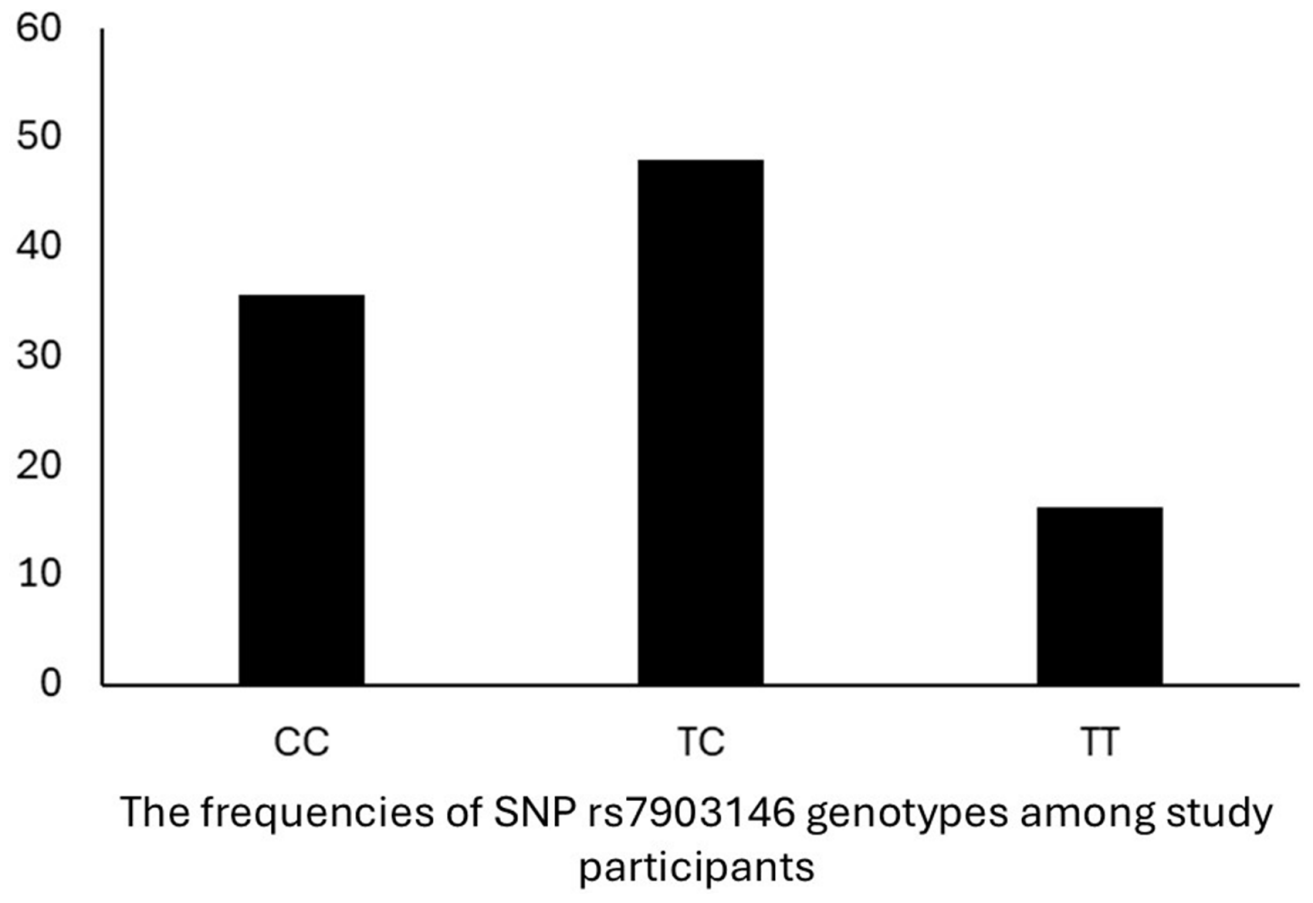
The frequencies of SNP rs7903146 genotypes among study participants.

The frequencies of SNP rs7903146 genotypes among study participants were 35.6, 48.1, and 16.2% for CC, CT, and TT, respectively (Figure 1). The association between *TCF7L2* SNP rs7903146 genotypes, and factors that may contribute to MetS are shown in Table 3. Only a marginal association was observed between the SNP and WC (*p* = 0.05). Carriers of TT genotype had the highest WC (83.5 ± 20.1 cm) compared to CC (80.0 ± 14.2 cm) and TC (77.9 ± 13.9 cm) genotypes. Table 4 instead shows the association between rs7903146 SNP genotypes and participants’ dietary intake. A significant association was observed between SNP and intake of both total energy (*p* = 0.04) and SFA (*p* = 0.005). Carriers of the TT genotype had the highest total energy intake (TEI) (3606.9 ± 1554.7Kcal) compared to those of the CC (3598.3 ± 1459.9 Kcal) and TC (3202.6 ± 1363.9) genotypes. In addition, carriers of the TT genotype had the highest intake of SFA (66.8 ± 52.0 g) compared to carriers of the CC (52.2 ± 36.6 g) and TC (52.9 ± 39.4 g) genotypes.

**Table 3:** Association between *TCF7L2* gene SNP rs7903146 and common MetS risk factors (additive model).

	CC (*n* = 94)	TC (*n* = 127)	TT (*n* = 43)	*P* value
BMI (kg\m^2^)	25.6 ± 5.9	24.5 ± 5.9	26.2 ± 8.9	0.11
Waist circumference (cm)	80.0 ± 14.2	77.9 ± 13.9	83.5 ± 20.1	0.05
Hip circumference (cm)	103.3 ± 15.3	101.9 ± 12.2	105.8 ± 18.8	0.27
Systolic blood pressure (mmHg)	125.7 ± 14.9	126.2 ± 15.4	130.2 ± 19.4	0.71
Diastolic blood pressure (mmHg)	75.4 ± 10.5	74.4 ± 9.4	77.0 ± 11.7	0.59
Total cholesterol (mg\dl)	174.4 ± 32.9	170.8 ± 36.8	173.3 ± 33.6	0.85
High-density lipoprotein cholesterol (mg\dl)	49.7 ± 10.0	50.4 ± 11.9	49.1 ± 11.7	0.86
Low-density lipoprotein cholesterol (mg\dl)	111.1 ± 27.1	106.7 ± 28.9	110.9 ± 27.0	0.54
Triglycerides (mg\dl)	67.7 ± 27.7	66.1 ± 27.2	66.3 ± 28.6	0.86
Glucose (mg\dl)	84.4 ± 10.0	84.8 ± 13.1	90.3 ± 26.2	0.39
Insulin (μIU/mL)	13.6 ± 8.1	14.4 ± 12.4	14.8 ± 18.7	0.41

Data are presented as mean ± SD. *p* values were tested using univariate linear regression; age, sex, smoking, and physical activity were adjusted for in analysis and body mass index was added when testing for blood pressure, lipid profile, glucose, and insulin.

**Table 4:** Association between *TCF7L2* gene SNP rs7903146 and subjects dietary intake (additive model).

	CC (*n* = 96)	TC (*n* = 128)	TT (*n* = 44)	*P* value
Energy (calorie)	3598.3 ± 1459.9	3202.6 ± 1363.9	3606.9 ± 1554.7	0.04
Energy (kilojoules)	15032.1 ± 6096.7	13366.1 ± 5696.0	15036.7 ± 6482.0	0.04
Protein (g)	241.1 ± 351.0	293.4 ± 407.8	378.7 ± 547.0	0.10
Carbohydrates (g)	403.6 ± 157.6	365.2 ± 155.9	403.2 ± 163.9	0.96
Total fat (g)	147.2 ± 88.1	142.3 ± 94.0	174.0 ± 126.8	0.11
Cholesterol (g)	673.6 ± 434.3	665.6 ± 505.8	669.3 ± 414.1	0.56
Saturated fatty acid (g)	52.2 ± 36.6	52.9 ± 39.4	66.8 ± 52.0	0.005
Monounsaturated fatty acid (g)	46.6 ± 23.3	40.1 ± 20.3	47.7 ± 25.9	0.48
Polyunsaturated fatty acid (g)	28.5 ± 15.4	25.9 ± 14.8	31.3 ± 20.2	0.79
Omega-3 (g)	1.0 ± 0.8	0.8 ± 0.7	1.1 ± 0.9	0.68
Omega-6 (g)	5.7 ± 4.8	4.7 ± 4.1	6.0 ± 4.8	0.83
Total dietary fiber (g)	30.8 ± 14.4	26.4 ± 13.2	26.8 ± 12.1	0.05

Data are presented as mean ± SD. *P* values was tested using univariate linear regression; age, sex, smoking, and physical activity, body mass index, and total calories were adjusted for in analysis.

The interaction between the *TCF7L2* rs7903146 SNP and TEI on factors that may contribute to MetS was analyzed (Table 5). Only a nearly significant interaction between SNP and TEI on insulin levels was evident (*p* = 0.04). Low TEI was associated with greater reduction in insulin levels among the TT genotype (−5.3 ± 3.5; confidence interval [CI] -12.4 − 1.7) compared to CC (−2.4 ± 3.1; 95% CI -8.4-3.5), and TC (−4.7 ± 2.8; 95% CI -10.3-0.8). The interaction between SNP and intake of carbohydrate, total fat, SFA, MUFA, and PUFA on factors that may contribute to MetS was also analyzed, with no significant found in the interaction models (Supplementary Tables 1–5).

**Table 5:** Interaction between *TCF7L2* gene SNP rs7903146 and total energy intake on common MetS risk factors (additive model).

	CC (*n* = 96)		TC (*n* = 128)		TT (*n* = 44)		*P*_interaction_
	Low calories	High calories	Low calories	High calories	Low calories	High calories	
BMI (kg\m^2^)	−1.7 ± 1.6	−2.4 ± 1.5	−2.7 ± 1.5	−3.6 ± 1.5	−2.5 ± 1.9	Reference	0.27
Waist circumference (cm)	−3.5 ± 3.5	−2.0 ± 3.2	−4.9 ± 3.3	−5.7 ± 3.3	1.1 ± 4.1	Reference	0.27
Hip circumference (cm)	−2.5 ± 3.8	−1.8 ± 3.5	−3.9 ± 3.5	−3.3 ± 3.5	1.1 ± 4.5	Reference	0.80
Systolic blood pressure (mmHg)	−2.2 ± 3.4	−1.8 ± 3.2	−2.2 ± 3.2	−1.4 ± 3.2	0.3 ± 4.1	Reference	0.98
Diastolic blood pressure (mmHg)	1.1 ± 2.4	1.5 ± 2.2	0.3 ± 2.2	0.9 ± 2.3	4.4 ± 2.9	Reference	0.64
Total cholesterol (mg\dl)	−9.1 ± 9.1	3.3 ± 8.5	−10.7 ± 8.4	5.6 ± 8.6	−4.3 ± 10.7	Reference	0.12
High-density lipoprotein cholesterol (mg\dl)	−1.6 ± 2.7	−1.1 ± 2.5	−1.3 ± 2.5	0.9 ± 2.5	−1.5 ± 3.2	Reference	0.87
Low-density lipoprotein cholesterol (mg\dl)	−6.6 ± 7.3	3.1 ± 6.8	−8.8 ± 6.7	2.5 ± 6.9	−2.5 ± 8.6	Reference	0.25
Triglycerides (mg\dl)	−5.7 ± 6.9	6.8 ± 6.4	−3.9 ± 6.3	6.1 ± 6.5	−3.4 ± 8.1	Reference	0.07
Glucose (mg\dl)	−4.5 ± 4.1	−1.6 ± 3.8	−2.5 ± 3.7	−1.4 ± 3.8	4.2 ± 4.8	Reference	0.58
Insulin (μIU/mL)	−2.4 ± 3.1	−1.9 ± 2.8	−4.7 ± 2.8	2.5 ± 2.8	−5.3 ± 3.5	Reference	0.04

Data are presented as Beta coefficient ± stander error. *p* values were tested using univariate linear regression; age, sex, smoking, and physical activity were adjusted for in analysis and body mass index was added when testing for blood pressure, lipid profile, glucose, and insulin.

## 4 Discussion

To the best of our knowledge, this is the first study to investigate the interactions among *TCF7L2* genotypes, dietary intake, and factors that may contribute to MetS in Saudi Arabian adults. A marginally significant association was observed between SNP rs7903146 and WC, and carriers of the TT genotype had the highest WC. Significant associations were observed between *TCF7L2* gene SNP rs7903146 and TEI and SFA intake. TT appears to be risk genotype, with carriers having the highest TEI and SFA intakes. An interaction was reported between SNP rs7903146 and TEI on insulin levels; however, it was not significant following Bonferroni correction. The low TEI intake was associated with decreased insulin levels in all genotypes.

Several meta-analysis studies demonstrated strong association between *TCF7L2* rs7903146 and increased risk of T2D different ethnic groups (39, 40). Also, a meta-analysis conducted among Arab patients with T2D found that *TCF7L2* polymorphism rs7903146 was associated increased odds of T2D by 1.34 (95% CI 1.27–1.41) (41). In line with our association results, a previous study conducted on 359 patients with T2D and 351 healthy controls in Saudi Arabia did not reveal an association between SNP rs7903146 and T2D risk. Also, genotype frequency was comparable to that in a previous study (19). However, a larger case–control study conducted on 1,166 patients with T2D, and 1,235 healthy Saudi Arabian participants demonstrated a significant association between the SNP rs7903146 and T2D, with T allele carriers showing an increased risk of the disease (18). The lack of association in the current study may be due to its cross-sectional design, including a relatively small sample size with participants of younger ages; the two previous studies were case–control studies (18, 19). Previous studies have only examined the risk of T2D; in our study, several factors that may contribute to MetS were analyzed aside from T2D risk. In addition, SNP rs7903146 was associated with WC in the Algerian population (n = 751), where T allele carriers had low WC (42), however in our study T allele carriers had increased WC. The SNP rs7903146 affects insulin secretion and *β*-cell function and the TT allele is associated with progression from impaired glucose tolerance to T2D. Such impairment increases fat accumulation which in turn increases WC (43). A previous report indicated that a reduction in anthropometric measurements by >5%, including WC, was clinically meaningful in weight management and reduced cardiovascular risk factors (44). Based on a literature search, no previous study has reported on the association between SNP rs7903146 and TEI or SFA, whose findings could be compared to the findings of the present study.

In contrast to our interaction finding, following a lifestyle intervention for 2 years that included energy intake reduction (fat <30%) and an increase in fiber intake, no association with changes in glucose or insulin sensitivity were observed for SNP rs7903146 risk allele carriers among 309 German participants with T2D; however, association with changes in BMI was observed (45). The differences could be attributed to study design, health status of participants, different populations, and different methods of assessing dietary intake. Evidence from randomized clinical trials that involved macronutrient modification (restricted energy intake and energy from fat) for weight loss demonstrated better glycemic control in the *TCF7L2* genotype, especially among T allele carriers (46, 47). Therefore, improvement in glucose controls can lead to lower insulin levels. A reduction in insulin levels by 10% has clinical significance for improvement in insulin sensitivity (48). Another intervention study used the genetic risk scores of six SNPs, including rs7903146, in Mexican adults with MetS. The intervention protocol involved consuming low amounts of SFAs (< 7%) for 2.5 months (< 200 mg of cholesterol/day) and reducing the TEI by 25%. The results showed increased HDL-C levels in individuals with low genetic risk scores (49). However, in the present study, no interaction with HDL levels was observed. The relatively small sample size may hinder observation of significant interaction findings. Notably, gene-diet interactions are probabilistic rather than deterministic, which implies that it increases or decreases the likelihood of the traits/diseases, and it is influenced by other environmental factors, including smoking, sedentary lifestyle, and physical activity. Therefore, in our analysis, we adjusted for physical activity and smoking to minimize their effects.

Previous studies have reported interactions between SNP rs7903146 and macronutrients on T2D risk. Two studies reported the interaction between SNP rs7903146 and carbohydrates on HbA1c (50, 51). However, consistent with our findings, two larger cohorts did not observe interaction between SNP and carbohydrates intake on T2D risk (2, 52). Moreover, a study on healthy Lebanese adults has shown that SFA and rs7903146 of *TCF7L2* have interaction effects on BMI (53). In addition, SFA SNP had interaction effects on metabolic risk in a case–control study (*n* = 1754) (22). MUFA is another macronutrient that modulates the association between *TCF7L2* polymorphism and HbA1c concentration (48). Moreover, PUFA intake interacted with SNP rs7903146 on modulating postprandial TG levels (54).

The dietary pattern of the Saudi population is distinct as it is high in refined carbohydrates (such as rice and bread) and red meat, and low in fruit and vegetables, in comparison to the Mediterranean diet or other unhealthy dietary patterns such as the Western diet. The present study was conducted solely in the Saudi population. The findings might not be applicable to other ethnicities because of differences in genetic ancestry and dietary patterns between Saudi and other populations. Numerous factors could explain the lack of significant interactions in the present study when compared with other studies: the sample size in our study is relatively small for interaction investigations, and other studies have been case–control or intervention studies, whereas our study was a cross-sectional study. In addition, the participants in the present study were younger adults and healthy, either normal, overweight or obese, whereas the participants of other studies were older, and some recruited healthy and non-healthy participants. Moreover, the dietary intake assessment method differs as some studies used 24 h while our assessment was based on an FFQ, and differences in statistical analysis tests were used. Finally, the inconsistencies may reflect differences in the criteria employed to define studies, such as population size, study design, methods of assessing dietary intake, age, genetic heterogeneity, or the dietary environment of the populations studied.

However, the results of this study should be considered in light of the following limitations. The study was cross-sectional, which limited the ability to establish causality among *TCF7L2* SNP rs7903146, dietary intake, and factors that may contribute to MetS, in addition to the relatively small sample size and the inclusion of predominantly younger adults. Thus, future studies based on longitudinal design and larger sample sizes are recommended. Although in-person interviews using food photographs were conducted to collect dietary intake data, the FFQ method may be subject to recall response bias and social desirability bias, and it was limited by measurement errors, reliance on memory, and the number of food items included in the food list. It may also overestimate the energy intake. Consequently, future studies should use dietary biomarkers to address such shortcomings and better capture intra-individual variability in intake. In addition, considering the present study was conducted solely in the Saudi population, the findings might not be applicable to other ethnicities because of differences in genetic ancestry and dietary patterns between Saudi and other populations.

## 5 Conclusion

In conclusion, in this nutrigenetic study, the interactions between the *TCF7L2* gene and dietary intake on factors that may contribute to MetS among Saudi Arabian adults were investigated for the first time. TT carriers had risk associated with increasing WC, TEI, and SFA intake. *TCF7L2* SNP rs7903146 carriers would benefit from low TEI, as it has favorable effects on insulin levels. Future studies using longitudinal study design, intervention-based research, including larger and more diverse sample sizes, and addressing the cumulative effect of several genes using gene-risk score are warranted.

## Data Availability

The original contributions presented in the study are publicly available. This data can be found at https://doi.org/10.5061/dryad.6m905qgbg.
