# Supplementary material for: Interaction between the TCF7L2 gene and dietary intake on metabolic syndrome risk factors among Saudi Arabian adults
Source: Front Nutr. 2025 Mar 18;12:1513088. doi: 10.3389/fnut.2025.1513088 (PMC11960299; doi:10.3389/fnut.2025.1513088)
Supplement: Supplementary file 1 [file Table_1.docx]

**Supplementary table 1 Interaction between *TCF7L2* gene SNP rs7903146 and carbohydrates calorie on common MetS risk factors (additive model)**

|  | CC (n=96) | | TC (n=128) | | TT (n=44) | | P _interaction_ |
| --- | --- | --- | --- | --- | --- | --- | --- |
|  | Low calories | High calories | Low calories | High calories | Low calories | High calories |  |
| BMI (kg\m^2^) | -0.5± 1.6 | -0.8± 1.5 | -1.5± 1.5 | -2.0± 1.5 | 0.5± 1.9 | Reference | 0.42 |
| Waist circumference (cm) | -4.5± 3.4 | 0.3± 3.3 | -4.3± 3.2 | -4.7± 3.2 | 2.8± 4.1 | Reference | 0.08 |
| Hip circumference (cm) | -4.9± 3.7 | -1.1± 3.5 | -5.2± 3.5 | -3.4± 3.5 | -0.4± 4.4 | Reference | 0.62 |
| Systolic blood pressure (mmHg) | -1.3± 3.4 | -2.6± 3.2 | -1.8± 3.1 | -2.1± 3.2 | 0.2± 4.1 | Reference | 0.95 |
| Diastolic blood pressure (mmHg) | 0.4± 2.4 | 0.3± 2.3 | -0.4± 2.2 | -0.4± 2.3 | 2.1± 2.8 | Reference | 0.93 |
| Total cholesterol (mg\dl) | -3.7± 9.1 | 0.5± 8.6 | -8.1± 8.4 | 2.6± 8.6 | -3.2± 10.7 | Reference | 0.66 |
| High-density lipoprotein cholesterol (mg\dl) | -1.4± 2.6 | -1.3± 2.5 | -1.0± 2.4 | 0.1± 2.5 | -1.7± 3.1 | Reference | 0.95 |
| Low-density lipoprotein cholesterol (mg\dl) | -2.2± 7.2 | 1.1± 6.8 | -6.6± 6.7 | 0.6± 6.9 | -1.1± 8.5 | Reference | 0.65 |
| Triglycerides (mg\dl) | -1.4± 6.8 | 4.0± 6.5 | -2.9± 6.3 | 4.4± 6.5 | -3.2± 8.1 | Reference | 0.58 |
| Glucose (mg\dl) | -2.4± 4.0 | -0.9± 3.8 | -1.1± 3.7 | -0.5± 3.8 | 7.0± 4.7 | Reference | 0.67 |
| Insulin (µIU/mL) | -1.9± 3.0 | -1.3± 2.8 | -3.9± 2.7 | 2.3± 2.8 | -4.2± 3.5 | Reference | 0.16 |

Data were presented as Beta coefficient± stander error.

P values were tested using univariate linear regression; age, sex, smoking, and physical activity were adjusted for in analysis and body mass index was added when testing for blood pressure, lipid profile, glucose, and insulin.

**Supplementary table 2 Interaction between TCF7L2 gene SNP rs7903146 and total fat calorie on common MetS risk factors (additive model)**

|  | CC (n=96) | | TC (n=128) | | TT (n=44) | | P _interaction_ |
| --- | --- | --- | --- | --- | --- | --- | --- |
|  | Low calories | High calories | Low calories | High calories | Low calories | High calories |  |
| BMI (kg\m^2^) | -1.2± 1.6 | -3.1± 1.6 | -2.8± 1.5 | -3.8± 1.5 | -2.5± 1.9 | Reference | 0.12 |
| Waist circumference (cm) | -2.7± 3.5 | -3.4± 3.4 | -4.9± 3.3 | -6.6± 3.3 | 0.1± 4.1 | Reference | 0.31 |
| Hip circumference (cm) | -3.9± 3.8 | -3.6± 3.6 | -5.1± 3.6 | -5.3± 3.6 | -2.2± 4.3 | Reference | 0.76 |
| Systolic blood pressure (mmHg) | -3.3± 3.4 | -3.2± 3.3 | -2.9± 3.2 | -3.4± 3.3 | -2.3± 4.0 | Reference | 0.98 |
| Diastolic blood pressure (mmHg) | 1.3± 2.4 | -0.3± 2.3 | -0.3± 2.3 | -0.4± 2.3 | 2.1± 2.8 | Reference | 0.84 |
| Total cholesterol (mg\dl) | -2.6± 9.2 | 1.8± 8.9 | -7.0± 8.7 | 4.1± 8.9 | -0.5± 10.6 | Reference | 0.66 |
| High-density lipoprotein cholesterol (mg\dl) | -1.4± 2.7 | -0.7± 2.6 | -0.6± 2.5 | 0.3± 2.6 | -0.9± 3.1 | Reference | 0.97 |
| Low-density lipoprotein cholesterol (mg\dl) | -1.1± 7.3 | 1.9± 7.1 | -5.4± 7.0 | 1.5± 7.1 | 0.8± 8.4 | Reference | 0.81 |
| Triglycerides (mg\dl) | -0.9± 6.9 | 3.8± 6.6 | -4.5± 6.5 | 7.1± 6.6 | -2.7± 7.9 | Reference | 0.23 |
| Glucose (mg\dl) | -9.8± 4.1 | -7.3± 3.9 | -8.1± 3.8 | -7.2± 3.9 | -7.3± 4.6 | Reference | 0.51 |
| Insulin (µIU/mL) | -2.5± 3.1 | -2.3± 3.0 | -2.8± 2.9 | -1.0± 2.9 | -5.5± 3.5 | Reference | 0.50 |

Data were presented as Beta coefficient± stander error.

P values were tested using univariate linear regression; age, sex, smoking, and physical activity were adjusted for in analysis and body mass index was added when testing for blood pressure, lipid profile, glucose, and insulin.

**Supplementary table 3 Interaction between TCF7L2 gene SNP rs7903146 and saturated fat calorie on common MetS risk factors (additive model)**

|  | CC (n=96) | | TC (n=128) | | TT (n=44) | | P _interaction_ |
| --- | --- | --- | --- | --- | --- | --- | --- |
|  | Low calories | High calories | Low calories | High calories | Low calories | High calories |  |
| BMI (kg\m^2^) | -1.7± 1.6 | -3.0± 1.5 | -3.1± 1.5 | -3.7± 1.5 | -3.1± 1.9 | Reference | 0.25 |
| Waist circumference (cm) | -1.9± 3.5 | -2.5± 3.3 | -4.4± 3.3 | -5.3± 3.3 | 1.9± 4.1 | Reference | 0.31 |
| Hip circumference (cm) | -4.1± 3.7 | -2.6± 3.6 | -5.4± 3.5 | -4.2± 3.5 | -1.5± 4.4 | Reference | 0.74 |
| Systolic blood pressure (mmHg) | -1.9± 3.4 | -20.8± 3.2 | -1.1± 3.32 | -1.4± 3.2 | 1.7± 4.1 | Reference | 0.96 |
| Diastolic blood pressure (mmHg) | 0.03± 2.4 | -0.8± 2.3 | -1.4± 2.3 | -0.9± 2.3 | 0.3± 2.9 | Reference | 0.95 |
| Total cholesterol (mg\dl) | -5.1± 9.1 | 0.2± 8.7 | -9.4± 8.6 | 2.1± 8.7 | -4.8± 10.8 | Reference | 0.61 |
| High-density lipoprotein cholesterol (mg\dl) | -1.5± 2.6 | -1.6± 2. 5 | -1.2± 2.5 | -0.1± 2.5 | -2.1± 3.1 | Reference | 0.95 |
| Low-density lipoprotein cholesterol (mg\dl) | -3.6± 7.2 | 1.2± 7.0 | -7.4± 6.9 | 0.1± 6.9 | -2.4± 8.6 | Reference | 0.72 |
| Triglycerides (mg\dl) | -0.1± 6.8 | 4.6± 6.5 | -3.4± 6.5 | 6.8± 6.5 | -1.3± 8.1 | Reference | 0.35 |
| Glucose (mg\dl) | -12.0± 3.9 | -8.2± 3.8 | -9.6± 3.7 | -8.7± 3.8 | -11.1± 4.7 | Reference | 0.13 |
| Insulin (µIU/mL) | -2.3± 3.1 | -1.9± 2.9 | -2.5± 2.9 | -0.7± 2.9 | -5.2± 3.6 | Reference | 0.39 |

Data were presented as Beta coefficient± stander error.

P values were tested using univariate linear regression; age, sex, smoking, and physical activity were adjusted for in analysis and body mass index was added when testing for blood pressure, lipid profile, glucose, and insulin.

**Supplementary table 4 Interaction between TCF7L2 gene SNP rs7903146 and monounsaturated fat calorie on common MetS risk factors (additive model)**

|  | CC (n=96) | | TC (n=128) | | TT (n=44) | | P _interaction_ |
| --- | --- | --- | --- | --- | --- | --- | --- |
|  | Low calories | High calories | Low calories | High calories | Low calories | High calories |  |
| BMI (kg\m^2^) | -1.3± 1.6 | -2.7± 1.5 | -2.8± 1.5 | -3.6± 1.5 | -2.7± 2.0 | Reference | 0.18 |
| Waist circumference (cm) | -3.9± 3.5 | -1.8± 3.2 | -4.6± 3.2 | -6.3± 3.3 | 1.0± 4.2 | Reference | 0.25 |
| Hip circumference (cm) | -2.3± 3.8 | -2.1± 3.5 | -3.4± 3.5 | -4.1± 3.5 | 0.9± 4.5 | Reference | 0.79 |
| Systolic blood pressure (mmHg) | -4.1± 3.4 | -2.4± 3.1 | -3.1± 3.1 | -2.9± 3.2 | -2.3± 4.2 | Reference | 0.96 |

| Diastolic blood pressure (mmHg) | 0.4± 2.4 | 0.4± 2.2 | 0.1± 2.2 | -0.9± 2.3 | 2.4± 3.0 | Reference | 0.88 |
| --- | --- | --- | --- | --- | --- | --- | --- |
| Total cholesterol (mg\dl) | -8.1± 9.2 | 1.0± 8.4 | -11.1± 8.3 | 5.4± 8.6 | -6.2± 10.9 | Reference | 0.21 |
| High-density lipoprotein cholesterol (mg\dl) | -1.5± 2.7 | -0.1± 2.4 | -1.0± 2.4 | 2.0± 2.5 | -0.2± 3.2 | Reference | 0.71 |
| Low-density lipoprotein cholesterol (mg\dl) | -5.8± 7.3 | 0.2± 6.7 | -9.3± 6.7 | 1.1± 6.9 | -5.4± 8.8 | Reference | 0.39 |
| Triglycerides (mg\dl) | -4.2± 6.9 | 4.8± 6.3 | -3.9± 6.3 | 5.8± 6.5 | -4.4± 8.2 | Reference | 0.19 |
| Glucose (mg\dl) | -12.1± 4.1 | -8.2± 3.7 | -10.1± 3.7 | -7.3± 3.8 | -11.6± 4.8 | Reference | 0.09 |
| Insulin (µIU/mL) | -2.1± 3.1 | -1.1± 2.8 | -1.6± 2.8 | -0.5± 2.9 | -4.3± 3.7 | Reference | 0.51 |

Data were presented as Beta coefficient± stander error.

P values were tested using univariate linear regression; age, sex, smoking, and physical activity were adjusted for in analysis and body mass index was added when testing for blood pressure, lipid profile, glucose, and insulin.

**Supplementary table 5 Interaction between TCF7L2 gene SNP rs7903146 and polyunsaturated fat calorie on common MetS risk factors (additive model)**

|  | CC (n=96) | | TC (n=128) | | TT (n=44) | | P _interaction_ |
| --- | --- | --- | --- | --- | --- | --- | --- |
|  | Low calories | High calories | Low calories | High calories | Low calories | High calories |  |
| BMI (kg\m^2^) | -1.2± 1.6 | -1.4± 1.5 | -2.1± 1.5 | -2.6± 1.5 | -0.8± 1.9 | Reference | 0.47 |
| Waist circumference (cm) | -1.9± 3.5 | 0.1± 3.2 | -3.2± 3.2 | -3.6± 3.2 | 5.3± 4.1 | Reference | 0.18 |
| Hip circumference (cm) | -1.3± 3.7 | 0.1± 3.5 | -2.5± 3.5 | -1.5± 3.5 | 4.7± 4.4 | Reference | 0.68 |
| Systolic blood pressure (mmHg) | -3.3± 3.4 | -2.5± 3.2 | -3.2± 3.1 | -2.1± 3.2 | -1.6± 4.1 | Reference | 0.96 |
| Diastolic blood pressure (mmHg) | 1.5± 2.4 | 1.3± 2.2 | 0.7± 2.2 | 0.4± 2.2 | 4.5± 2.8 | Reference | 0.65 |
| Total cholesterol (mg\dl) | -3.8± 9.1 | 6.3± 8.5 | -4.4± 8.5 | 5.1± 8.6 | 4.0± 10.7 | Reference | 0.53 |
| High-density lipoprotein cholesterol (mg\dl) | -1.1± 2.6 | -0.3± 2.5 | 0.3± 2.5 | -0.02± 2.5 | -0.1± 3.1 | Reference | 0.99 |
| Low-density lipoprotein cholesterol (mg\dl) | -2.1± 7.2 | 5.6± 6.8 | -4.1± 6.7 | 3.3± 6.8 | 4.7± 8.5 | Reference | 0.57 |
| Triglycerides (mg\dl) | -4.8± 6.8 | 5.4± 6.4 | -3.8± 6.3 | 4.2± 6.4 | -4.6± 8.0 | Reference | 0.32 |
| Glucose (mg\dl) | -2.6± 4.0 | -0.3± 3.7 | -1.6± 3.7 | 0.6± 3.8 | 7.6± 4.7 | Reference | 0.44 |
| Insulin (µIU/mL) | -1.1± 3.1 | -1.4± 2.8 | -0.7± 2.8 | -1.2± 2.9 | -3.6± 3.6 | Reference | 0.82 |

Data were presented as Beta coefficient± stander error.

P values were tested using univariate linear regression; age, sex, smoking, and physical activity were adjusted for in analysis and body mass index was added when testing for blood pressure, lipid profile, glucose, and insulin.
